# MiR-659-3p inhibits osteosarcoma progression and metastasis by inhibiting cell proliferation and invasion via targeting *SRPK*1

**DOI:** 10.1186/s12885-022-10029-0

**Published:** 2022-08-29

**Authors:** Yubao Gong, Zheng-Ren Wei

**Affiliations:** 1 Department of Orthopaedics, the First Hospital of Jilin University, 1 Xinmin Street, Changchun, 130021 China; 2 Department of Pharmacology, Basic Medical School, Jilin University, 126 Xinmin Street, Changchun, 130021 China

**Keywords:** Osteosarcoma, Serine-arginine protein kinase 1 (SRPK1), microRNA, miR-659-3p, Metastasis

## Abstract

**Objective:**

Osteosarcoma is the most common primary bone cancer that affects mostly children and young adults. Despite the advances in osteosarcoma treatment, the long-term survival rate of metastatic patients has not significantly improved in the past few decades, thus demonstrating the need for novel therapeutic targets or methods to improve metastatic osteosarcoma treatment. In this study we aimed to elucidate the role of miR-659-3p and SRPK1 in osteosarcoma.

**Methods:**

We evaluated miR-659-3p and SRPK1 function in osteosarcoma cell proliferation, migration, and cell cycle progression in vitro by using gain- and loss-of-function strategies. The effect of miR-659-3p in tumor progression and metastasis was determined by *in vivo* mouse model.

**Results:**

We revealed that expression of miR-659-3p was significantly downregulated in osteosarcoma compared with normal bone cells and was inversely correlated with serine-arginine protein kinase 1 (*SRPK*1) expression. We proved that miR-659-3p targets 3’ UTR of *SRPK1* and negatively regulates *SRPK*1 expression in osteosarcoma cells via luciferase assay. *In vitro* studies revealed that gain of miR-659-3p function inhibited osteosarcoma cells growth, migration, and invasion by down-regulating *SRPK*1 expression. Inversely, inhibiting miR-659-3p in osteosarcoma cells promoted cell growth, migration, and invasion. Cell cycle profile analysis revealed that miR-659-3p inhibited osteosarcoma cells’ G1/G0 phase exit by down-regulating *SRPK*1 expression. By using an *in vivo* mouse model, we demonstrated that miR-659-3p inhibits osteosarcoma tumor progression and lung metastasis by inhibiting *SRPK*1 expression and potentially downstream cell proliferation, and epithelial-to-mesenchymal transition genes.

**Conclusions:**

This study demonstrated that miR-659-3p is a potential therapeutic method and SRPK1 is a potential therapeutic target for osteosarcoma treatment.

**Supplementary Information:**

The online version contains supplementary material available at 10.1186/s12885-022-10029-0.

## Introduction

Osteosarcoma is the most common primary bone cancer that affects mostly children and young adults, although it can occur at any age range [1]. In the recent years, the overall survival rate of osteosarcoma patients has greatly increased due to advances in the osteosarcoma treatment. However, in the recent decades, the long-term survival rate of osteosarcoma patients with metastatic tumors has remained at about 25% due to lack of effective treatment for metastatic osteosarcoma [2]. Thus, there is an urgent need to develop novel therapeutic targets for metastatic osteosarcoma treatment.

Fundamental processes in the mammalian gene expression include pre-mRNA splicing and alternative splicing, which are methods of generating protein diversity in mammalian cells. Most cellular processes, such as transcription, DNA replication, etc. depend on the proper function of spliceosome, because nearly all of the genes undergo pre-mRNA splicing and alternative splicing [3]. As an important unit of a spliceosome, the serine-arginine protein kinase 1 (SRPK1) phosphorylates splicing factors rich in serine/arginine domains (SR proteins) such as SRFS1 (serine/arginine-rich splicing factor 1) [4, 5]. By interacting with chaperone proteins, SRPK1 regulates the network for splicing, controlling the distribution of splicing factors within the nuclear [6, 7]. Therefore, SRPK1 is a critical player in pre-mRNA splicing and alternative splicing regulation, which affects almost all cellular processes such as proliferation, apoptosis, etc. The aberrant function of alternative splicing has been suggested as a key mechanism of carcinogenesis and linked to the hallmarks of several cancers [8, 9]. In fact, SRPK1 has been proved to play critical roles in many different cancers, such as gastric cancer [10, 11], lung cancer [12], breast cancer [13–17], prostate cancer [18–20], leukemia [21], hepatocellular carcinoma [22, 23], and so on. Targeting SRPK1 as a cancer treatment has been tested on cell lines and animal models (reviewed in [24]). However, whether SRPK1 plays any role in osteosarcoma is not clear.

MicroRNAs (miRNA) are a group of small non-coding-RNA molecules that negatively regulate gene expression at the post-transcriptional level by binding to mRNA [25, 26]. Numerous miRNAs have been found differentially expressed in osteosarcoma compared with normal bone tissue [27]. MiRNAs are known to play an essential role in the pathogenesis of osteosarcoma either as oncogenic or tumor suppressor agents because, depending on the targets, miRNA can regulate genes that play essential roles in cell proliferation, adhesion, invasion, migration, and metastasis, and apoptosis [28–30]. A few miRNAs has been identified to be play roles in osteosarcoma, such as miR-627-3p targets Pleiotrophin (PTN), which inhibits osteosarcoma cell proliferation and metastasis [31]. In addition, a classical tumor suppressor known as miR-34a has been found to inhibits osteosarcoma through multiple targets [32, 33]; MiR-199a-3p was found to be able to inhibit osteosarcoma as well by targeting Adenylate Kinase 4 (AK4) [34], and so on. Therefore, functional assessment of miRNA can be very important to not only determine its role in osteosarcoma pathogenesis and malignancy but also develop possible therapeutic method for osteosarcoma treatment [28, 35].

Taken together, elucidating whether SRPK1 plays a role in osteosarcoma progression and metastasis may provide novel osteosarcoma novel treatment target, and identification of novel miRNAs target SRPK1 in osteosarcoma may also provide a new therapeutic method for osteosarcoma treatment. In this study, we identified miR-659-3p as a negative regulator of *SRPK*1 gene in osteosarcoma cells. For the first time, we reported that miR-659–39 inhibits osteosarcoma cells growth *in vitro* and inhibits osteosarcoma tumor progression and metastasis *in vivo* by targeting *SRPK*1 gene expression. These findings may have established foundations for osteosarcoma treatment.

## Material and methods

### miRNA expression dataset and analysis

Non-coding RNA profiling data GSE28425 [36] was downloaded from Gene Expression Omnibus website: https://www.ncbi.nlm.nih.gov/geo. The GEO_2_R interactive tool was used to determine differential expression between osteosarcoma and control groups by calculating log base 2 of fold change (log_2_(FC)). Significance in fold change was determined from the adjusted p-value parameter calculated by the GEO_2_R interactive tool.

### Osteosarcoma cell lines and culture

The osteosarcoma cell lines, HOS, U-2 OS, MG-63, Saos-2, and normal osteoblast cell line hFOB1.19 were purchased from ATCC (www.ATCC.org) and maintained in our laboratory. The HOS and MG-63 cells were cultured in Eagle’s Minimum Essential Medium (Hyclone) supplied with 10% FBS (Gibco), 1% Penicillin**–**Streptomycin (Solarbio) at 37˚C 5% CO_2_. The Saos-2 and U-2OS cells were cultured in DMEM medium (Hyclone) containing 10% FBS and 1% Penicillin–Streptomycin at 37˚C 5% CO_2_. The normal osteoblast cell line hFOB1.19 cells were cultured in DMEM/F12 medium (Gibco) containing 10% FBS and 1% Penicillin–Streptomycin at 37˚C 5% CO_2_.

### Cell transfection

For transfecting the HOS or Saos-2 cells, the cells were plated in 6-well plates at the density of 3X10^5^ cells/well and cultured at 37˚C, 5% CO_2_ for 16 h. The cells were then transfected with irrelevant scramble miRNA as control (5’-CAGUACUUUUGUGUAGUACAA-3’, 100 pmol/well) or miR-659 mimic (5’-CUUGGUUCAGGGAGGGUCCCCA-3’, 100 pmol/well), or miR-659-3p inhibitor (5’-UGGGGACCCUCCCUGAACCAAG-3’, 100 pmol/well) by using Lipofectamine 2000 (Life technologies) and following manufacturer instructions. To overexpress *SRPK*1, the cells were transfected with expression vector pcDNA3.1 containing *SRPK*1 cDNA sequence (NM_003137.5) or empty pcDNA3.1 vector control (both were transfected with 1.5 μg/well). The overexpression of miR-659-3p or *SRPK*1 was confirmed by real-time PCR and/or Western blot.

### RNA isolation and Real-Time PCR

Total RNA was isolated from cells or tumor tissues by placing the cells or tissues in 1 ml Tryzol reagent (Invitrogen) and homogenized with Fluko homogenizer for 20 s. Total RNA was then isolated by following Tryzol reagent protocol. One microgram of total RNA from each sample was used to synthesize first strand cDNA by using RevertAid First Strand cDNA Synthesis Kit (Fermentas) and following the kit protocol. The first strand cDNA samples were used as a temperate for real-time PCR to quantify the expression of mRNA by using SYBR green (Thermofisher) and gene-specific primers (listed in Table 1). Additionally, *GAPDH* was used as an internal control. For quantification of miRNA, 2 μg of total RNA was used for poly-A tailing reaction by using New England BioLabs Poly-A polymerase. The RNA was then reverse transcribed into cDNA by using universal RT primer (5’-GCAGATCGTCAGAATTCCAGGC(T)_20_VN-3’, V = A,C,G,N = A,C,G,T) and SuperScript III reverse transcriptase (Life Technologies). Real-time PCR was then performed to quantify miRNA by using universal primer 5’-GCAGATCGTCAGAATTCCAG-3’ and miR-659-3p specific primers (Table 1). Additionally, small non-coding RNA U6 was used as an internal control.

**Table 1:** Real-time PCR primers sequence

Gene name	Forward primer sequence	Reverse primer sequence
miR-659-3p	TTGGTTCAGGGAGGGTCCCCA	GCAGATCGTCAGAATTCCAG
U6	CTCGCTTCGGCAGCACA	AACGCTTCACGAATTTGCGT
*SRPK*1	TGATACAGAGGGTGGTGC	TTTGGGAGCTTAGGAAAC
GAPDH	AATCCCATCACCATCTTC	AGGCTGTTGTCATACTTC

### Cell proliferation assay with Cell Counting Kit-8 (CCK8)

Cell growth assays were performed as reported before [28]. Briefly, 3000 transfected HOS or Saos-2 osteosarcoma cells were plated in each well of 96-well plates in complete growth medium. CCK8 (SAB) was diluted in serum-free medium (1:10). After the cells were further cultured at 37˚C, 5% CO_2_ for 12, 24, 48, and 72 h, 100 μl of diluted CCK8 was added to each well and incubated at 37˚C, 5% CO_2_ for 1 h. At each time point, OD 450 nm was measured in a plate reader (Pulang New Technology, Beijing).

### Cell migration assay

To test the migration capability of the cells, 8X10^5^ transfected HOS or Saos-2 osteosarcoma cells were plated in each well of 6-well plates in complete growth medium and cultured till confluence. The growth medium was then removed, and a gap of the cells was created by scratching the cells with a 1000 μl tip. The cells were then washed with PBS to remove the loose cells from the wells, and the cells were further cultured in complete growth medium for 12 and 24 h. The size of the gap was recorded by taking pictures of the gaps at each time point.

### Transwell cell invasion assay

Osteosarcoma cell invasion capability was assayed in transwells (Corning). Briefly, the transfected HOS or Saos-2 cells were starved in serum-free medium for 24 h. The cells were then plated in the upper chamber of the transwells at the density of 5X10^4^/well in 200 μl serum-free medium and inserted the upper chamber into the lower chamber containing 700 μl growth medium containing 10% FBS. The cells were cultured at 37˚C, 5% CO_2_ for 24 h. The cells in the lower chamber were fixed with 4% formalin at room temperature for 10 min after removing the upper chamber and growth medium from the wells. The cells were then washed with PBS and stained with 0.5% Crystal Violet at room temperature for 30 min and washed with PBS 3 times. The numbers of cells migrated into the lower chamber were scored by taking photosof the stained cells under microscope and counting the number of cells in each picture.

### Dual luciferase assay

The whole 3’ UTR sequence of *SRPK*1 gene (NCBI accession number: NM_003137) was obtained from NCBI library (Supplemental file 1). A 2288 bp wild-type (wt) *SRPK*1 3’ UTR DNA fragment and a putative miR-659-3p binding sites eliminated mutant 3’ UTR DNA fragment (Supplemental File 1) were synthesized (General Bio, Shanghai) and inserted into the 3’ of luciferase gene in the pGL3-promoter-Luc2 vector (Promega) by using Xba I restriction site. The result plasmids (called pGL3-wt and pGL3-mut hereafter) were sequenced to confirm the right orientation and sequences. Log phase HOS osteosarcoma cells were plated in 6-well plates at the density of 3 X 10^5^/well and cultured at 37˚C, 5% CO_2_ for 24 h. The cells were then co-transfected with combination of a, 1.5 μg of pGL3-wt, 20 ng of pRL-TK (Renilla luciferase control reporter vector, Promega), and 100 pmol of scramble miRNA; b, 1.5 μg of pGL3-wt, 20 ng of pRL-TK, and 100 pmol of miR-659-3p; c, 1.5 μg of pGL3-mut, 20 ng of pRL-TK, and 100 pmol of miR-659-3p, by using Lipofectamine 2000 and following the manufacturer’s protocol. The cells were washed with PBS and lysed with lysis buffer 48 h after transfection. Luciferase activity was analyzed with a dual luciferase assay kit (Promega).

### Western blot analysis

Total protein was extracted from the cells in culture or tumor tissue. Tumor tissues (about 20 mg of each sample) were washed with ice-cold PBS and were homogenized in RIPA buffer with a homogenizer on ice. Homogenized tumor tissues were then centrifuged at 12,000 RPM, 4ºC to remove the debris. The protein concentration of the supernatant was measured with Pierce BCA Protein Assay Reagent A (Thermo Fisher Scientific US, Cat# 23223). The cells cultured on the plate were washed with ice-cold PBS twice and directly lysed in RIPA buffer. Cell lysates were centrifuged at 12,000 RPM, 4ºC to remove the debris. Approximately 25 μg (from cells) or 50 μg (from tissue) of total protein was loaded into each well and separated by sodium dodecyl sulphate–polyacrylamide gel (SDS-PAGE) electrophoresis in a 10% polyacrylamide gel and transferred to immunoblot nitrocellulose (NC) membranes (Millipore). The membranes were sometimes stained with ATX Ponceau S red staining solution (Sigma Cat# 09189) to ensure the proteins were transferred. The target protein area was then cut from the whole membranes. The membranes were incubated with primary antibody against *SRPK*1 (Abcam, Cat# ab 90527, 1:500), PCNA (Abcam, Cat# ab92552, 1:1000), Ki67 (Abcam, Cat# ab16667, 1:2000), E-Cadherin (Cell Signaling Technologies, Cat# 14472, 1:1000), N-Cadherin (Cell Signaling Technologies, Cat# 13116, 1:1000), or GAPDH (Proteintech Cat# 60004–1-1G, 1:5000), followed by incubation with horseradish peroxidase-conjugated secondary antibodies (Biyuntian, China, Cat# A0208 and A0216, both 1:2000). ECL Western blotting analysis system was used to detect the binding of primary and secondary antibodies.

### Cell cycle profile analysis

Cell cycle profile analysis was performed as reported before [28]. Briefly, transfected HOS or Saos-2 cells were trypsinized from culture dishes and washed with ice-cold PBS twice. The cells were fixed by resuspending in ice-cold 70% ethanol and stored at -20˚C for 24 h. The cells were then washed with ice-cold PBS twice and stained with 20 μg/ml propidium iodide (Cell Cycle and Apoptosis Analysis kit, Beyotime) in 0.1% Triton-X 100/PBS with 0.2 μg/ml RNase A at a final concentration of 2X10^6^ cells/ml at 37˚C for 30 min. After staining, the cells were kept on ice and subject to FACS analysis (Beckman CytoFLEX flow cytometer). The cell cycle profile data was analyzed by using FlowJo software.

### Cell apoptosis assay

To evaluate whether miR-659-3p promotes osteosarcoma cells apoptosis, we utilized relative low miR-659-3p expression HOS cell line and relative high miR-659-3p expression Saos-2 cell line. HOS osteosarcoma cells were transfected with miR-659-3p mimic after overnight culture. Saos-2 cells were transfected with miR-659-3p inhibitor as described in cell transfection section. After 48 h culturing post transfection, the cells were briefly trypsinized and washed with FACS buffer (10% FBS in PBS), and then resuspended in Annixin V binding buffer and stained with FITC conjugated Annixin V (Biyuntian, Beijing, Cat# C1062) for 15 min. The cells were then washed once with FACS buffer and resuspended in 400 mL FACS buffer with propidium iodide (PI) and placed in the dark on ice. The cells were subjected to FACS analysis (Beckman CytoFLEX flow cytometer). The FACS data was analyzed by using FlowJo software.

### SRPK1 immunofluorescence staining

The protocol for immunofluorescence staining of the osteosarcoma sections was adapted from literature [37] and modified accordingly. Briefly, formalin fixed and paraffin-embedded osteosarcoma tissues were sectioned at 5–7 μm thickness. Sections then went through steps of deparaffinization, and incubation overnight at 4 °C with antibodies against SRPK1 (Proteintech, Cat# 14073–1-AP, 1:200 dilution). Sections were then incubated with an anti-rabbit secondary antibody conjugated with Alexa Fluor® 488 (Cell Signaling) after washed with washing buffer. The sections were mounted with mounting medium contains DAPI for counterstain. Images were acquired under fluorescent microscope.

### *In vivo* tumor model

All animal studies were approved by the Jilin University Institutional Animal Care and Use Committee. Six-week-old female Balb/c athymic nude mice were purchased from Shanghai Experimental Animal Center of Chinese Academy of Sciences. During the experiments, the mice were maintained in a special pathogen-free (SPF) grade facility. HOS osteosarcoma cells were cultured in 100 mm cell culture dishes until about 85% confluent and were collected from culture dishes with 0.05% Trypsin (Life technology). The cells were re-suspended in serum-free medium at 2X10^7^ cell/ml final concentration. Nude mice were randomly separated into 2 groups, with 6 mice in each group. Each mouse received 100 μl of cells injected subcutaneously (2 X 10^6^ cells/mouse). The mice were monitored closely every day. One group of mice were injected with 1 pmol of miR-659-3p mimic and the other group of mice were injected with scramble miRNA control via tail vein a week after tumor cells injection. MiR-659-3p or control miRNA was intravenously administered to the mice every 3 days for 3 total injections. The tumor sizes were measured when the tumors started to be visible and measured every 3 days. The volume of the tumor was calculated as indicated in the figure. Lung metastasis was determined by counting the number of tumors from 5 sections of the lungs cut at different depths and after H&E staining.

### Statistics analysis

All data presented in this manuscript are the mean ± standard deviation (SD). The comparison between two groups was analyzed with two-tailed Student’s T-Test. The comparisons among multiple groups were made with one-way or two-way ANOVA. P values less than 0.05 were considered statistically significant.

## Results

### MiR-659-3p targets 3’ UTR of *SRPK*1 gene and inhibits *SRPK*1 gene expression in osteosarcoma cells

**Fig.1:**
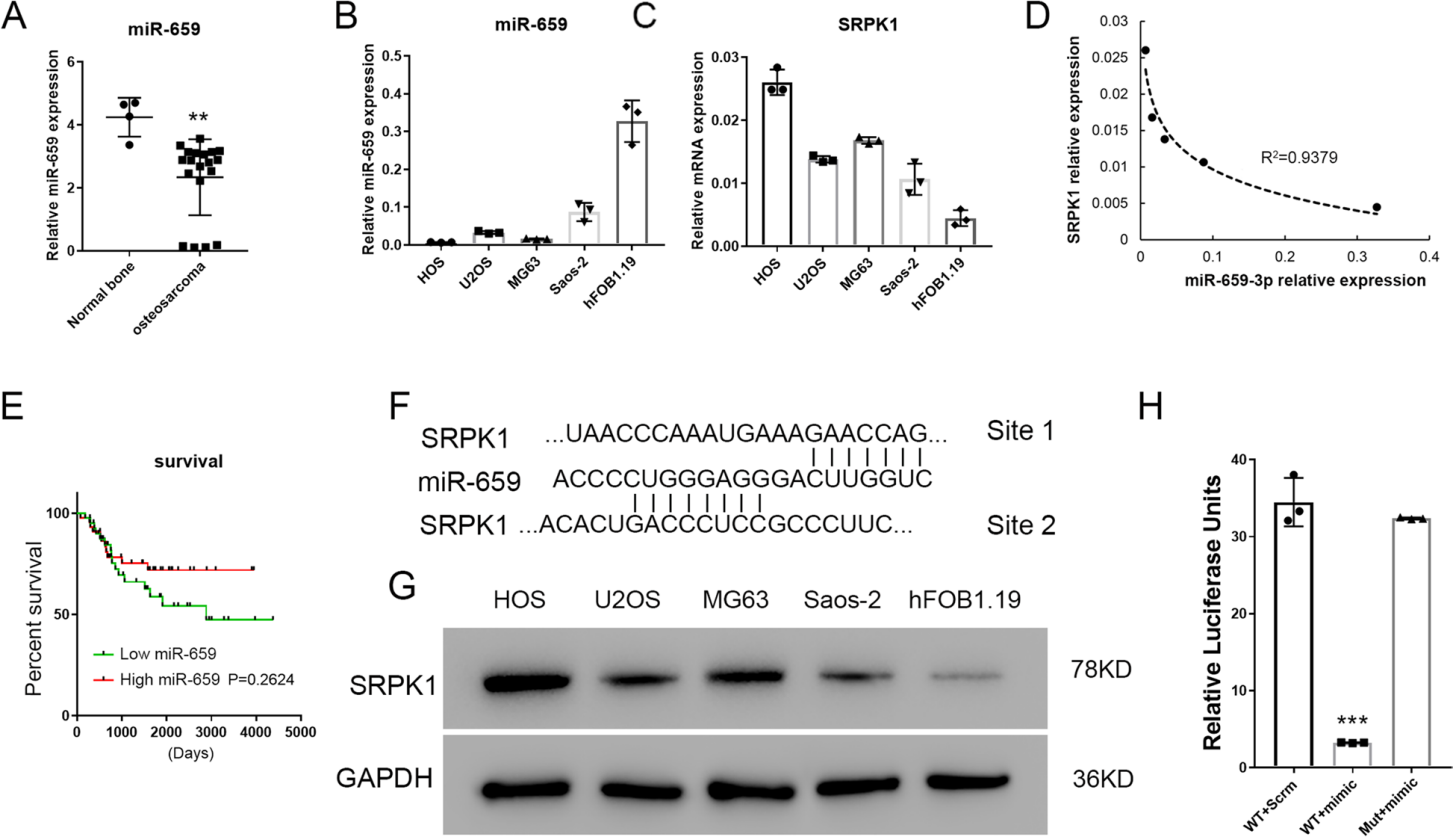
MiR-659-3p targets *SRPK*1 gene 3’UTR and negatively regulates *SRPK*1 expression in osteosarcoma cells. **A** MiR-659-3p was downregulated in human osteosarcoma compared with normal bone cells. The data was analyzed from the existing dataset from NCBI Gene Expression Omnibus GSE28423. **B** Quantitative real-time PCR verified that downregulated expression of miR-659-3p in human osteosarcoma cell lines compared with normal bone cells (hFOB1.19). **C** Quantitative real-time PCR showed that relative mRNA expression of *SRPK*1 is upregulated in osteosarcoma cell lines compared with normal bone cells. **D** Reverse correlation between miR-659-3p expression and SRPK1 mRNA expression in the cell lines assayed. **E** Worse prognosis observed in low miR-659-3p expression osteosarcoma patients compared with high miR-659-3p expression patients. **F** Alignment of miR-659-3p and *SRPK*1 3’ UTR showed potential targeting sites. **G** Western blot showed SRPK1 protein expression in human osteosarcoma cell lines, which confirmed the rough reverse correlation with miR-659-3p expression in the cell lines. SRPK1 and GAPDH western blots were performed on different gels. **H** Luciferase assay confirmed that miR-659-3p targets *SRPK*1 3’ UTR and negatively regulated its expression. ** indicates *P* < 0.01 and *** indicates *P* < 0.001 compared with control

In order to identify the miRNAs that target *SRPK*1, we analyzed differentially expressed microRNAs in the existing data set from NCBI Gene Expression Omnibus GSE28423, which was performed on 19 human osteosarcoma cell lines with 4 normal bone cell lines as control [36, 38]. We found that a total of 139 microRNAs were significantly down-regulated (logFC ≤ -1) in osteosarcoma compared to normal cells (Supplemental file 2). Meanwhile, we performed TargetScan search for the miRNAs that potentially target *SRPK*1 gene 3’ UTR and found 830 miRNAs that are predicted to bind to *SRPK*1 gene 3’ UTR. Among these 830 miRNAs, 11 of them were significantly down-regulated in the osteosarcoma based on the GSE28423 dataset analysis (Supplemental Fig. 1). A literature search found that 5 of these 11 miRNAs had not been reported to play roles in osteosarcoma by the time we analyzed the data. Thus, we performed preliminary screening of 5 novel miRNAs potentially targeting *SRPK*1 in osteosarcoma cell lines. We found that the miR-659-3p expression exhibited a reverse correlation with the expression of *SRPK*1 in osteosarcoma cell lines (Fig. 1A-D). Figure 1 A showed that miR-659-3p expression was downregulated by analyzing the expression data from dataset GSE28423. Real-Time PCR result revealed that the expression of miR-659-3p was significantly downregulated in osteosarcoma cell lines compared with normal bone (Fig. 1B). The mRNA expression of *SRPK*1 was significantly upregulated in the osteosarcoma cell lines compared to normal bone cell lines (Fig. 1C). A reverse correlation was found between the relative expression of miR-659-3p and *SRPK*1 mRNA in the cell lines (Fig. 1D). Interestingly, we found that low miR-659-3p expression is correlated with a worse prognosis in osteosarcoma patients (Fig. 1E), although it was not statistically significant due to the small numbers of patients. This is the only dataset that we can find available that has greater than 80 patients in the study. In order to further identify the miR-659-3p binding site(s) in *SRPK*1 3’ UTR, we performed a TargetScan search and sequence alignment analysis. TargetScan predicted one binding site (Fig. 1F Site 1) and through sequence alignment, and additional putative binding site was also found (Fig. 1F, Site 2). Taken together, we hypothesized that miR-659-3p targets *SRPK*1 gene, and negatively regulates *SRPK*1 gene expression. To prove this hypothesis, we first performed Western blot to assay the protein level expression in the osteosarcoma cell lines and FOB1.19 cells. Interestingly, SRPK1 expression at the protein level was also found to be downregulated in these cell lines (Fig. 1G). A luciferase reporter assay was further performed to confirm that miR-659-3p targets *SRPK*1 gene 3’UTR sequence and regulates *SRPK*1 gene expression. To eliminate the possibility of redundancy, both putative miR-659-3p binding sites were mutated in the 3’ UTR. The wild type and mutant *SRPK*1 gene 3’ UTR sequences were cloned into the 3’ of a luciferase reporter gene. If our hypothesis is correct, increase of miR-659-3p will target the 3’UTR sequence linked to luciferase RNA and will degrade luciferase RNA. Therefore, luciferase activity in the miR-659-3p mimic transfected cells will be reduced. Figure 1H showed that miR-659-3p mimic transfection significantly reduced luciferase activity in HOS cells with wild-type 3’UTR, but did not reduce luciferase activity when the miR-659-3p targeting sequence was mutated (Fig. 1H and Supplemental file 1). This data indicated that miR-659-3p specifically targets *SRPK*1 3’ UTR and negatively regulates *SRPK*1 gene expression.

### MiR-659-3p inhibits osteosarcoma cells proliferation and migration *in vitro*

**Fig. 2:**
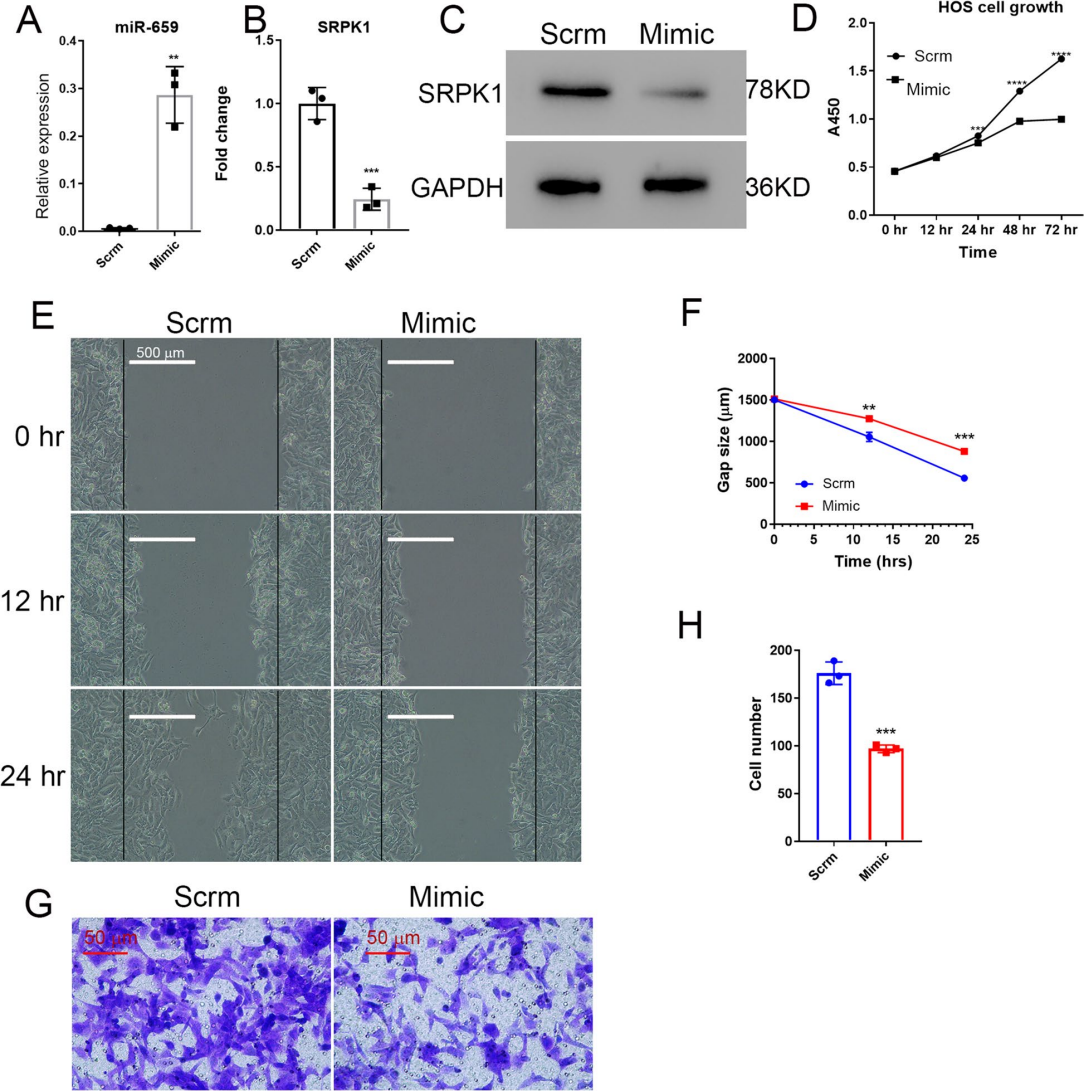
MiR-659-3p inhibits osteosarcoma cells growth, migration, and invasion. **A** Real-time PCR confirmed that transfection of miR-659-3p mimic increased miR-659-3p in osteosarcoma cells. **B** Real-time PCR showed downregulated *SRPK*1 expression by miR-659-3p mimic transfection. **C** Western blot confirmed SRPK1 expression was down-regulated on protein level. SRPK1 and GAPDH western blots were performed on different gels. **D** Transfection of miR-659-3p inhibited HOS cell growth. **E** Representative pictures of HOS osteosarcoma cells wound gap at 0, 12, and 24 h of culture. **F** Quantification of the HOS cell wound gap after 12 and 24 h culture (*n* = 3), which showed transfection of miR-659-3p mimic slowed HOS wound healing in cell culture dish. **G** Representative pictures of crystal violet stained HOS cells in the bottom chamber of transwells. **H** Quantification of HOS cells migrated through transwells to the bottom chamber (*n* = 3). ** indicates *P* < 0.01 and *** indicates *P* < 0.001 compared to Scramble control

*SRPK*1 plays a role in several cancers, although whether *SRPK*1 plays any role in osteosarcoma has not been established. Here we first asked the question of whether miR-659-3p inhibits osteosarcoma cells proliferation in vitro by negatively regulating *SRPK*1 expression. Transfection efficiency of miR-659-3p mimic in HOS cells was confirmed by real-time PCR, which showed above 40 fold increase of miR-659-3p (Fig. 2A). As expected, overexpression of miR-659-3p reduced expression of *SRPK*1 on mRNA level (Fig. 2B) and protein level by western blot (Fig. 2C). HOS osteosarcoma cells proliferation was significantly inhibited by miR-659-3p mimic transfection (Fig. 2D). We further assessed HOS osteosarcoma cells' migration capability. HOS osteosarcoma cells transfected with scramble miRNA or miR-659-3p mimic were cultured in 6-well plates till confluent. Cell gaps were created by scratching the cells with a 1 mL pipette tip. The cells were further cultured and the gap sizes were monitored. As shown in Fig. 1E and F, miR-659-3p mimic transfection significantly slowed HOS osteosarcoma cell migration. We further hypothesized that inhibiting *SRPK*1 expression by miR-659-3p transfection will inhibit HOS cell invasion. To test this hypothesis, we cultured HOS osteosarcoma cells either transfected with scramble miRNA or miR-659-3p mimic in the upper chamber of transwells. The cells migrated through transwells into the lower chamber of the plate after 24 h of culture were fixed, stained with crystal violet, and the cell number was counted after imaging. As shown in Fig. 2G and H, miR-659-3p significantly inhibited osteosarcoma cells invasion. HOS cells transfected with control scramble miRNA, 176 ± 6.8 cells were observed in each view in the lower chamber. However, only 97 ± 2.3 cells were observed in each view in the lower chamber transfected with miR-659-3p mimic. This data demonstrated that miR-659-3p inhibited *SRPK*1 expression and inhibited osteosarcoma cell proliferation, migration, and invasion.

### Inhibiting miR-659-3p promotes osteosarcoma cells proliferation and migration in vitro

**Fig. 3:**
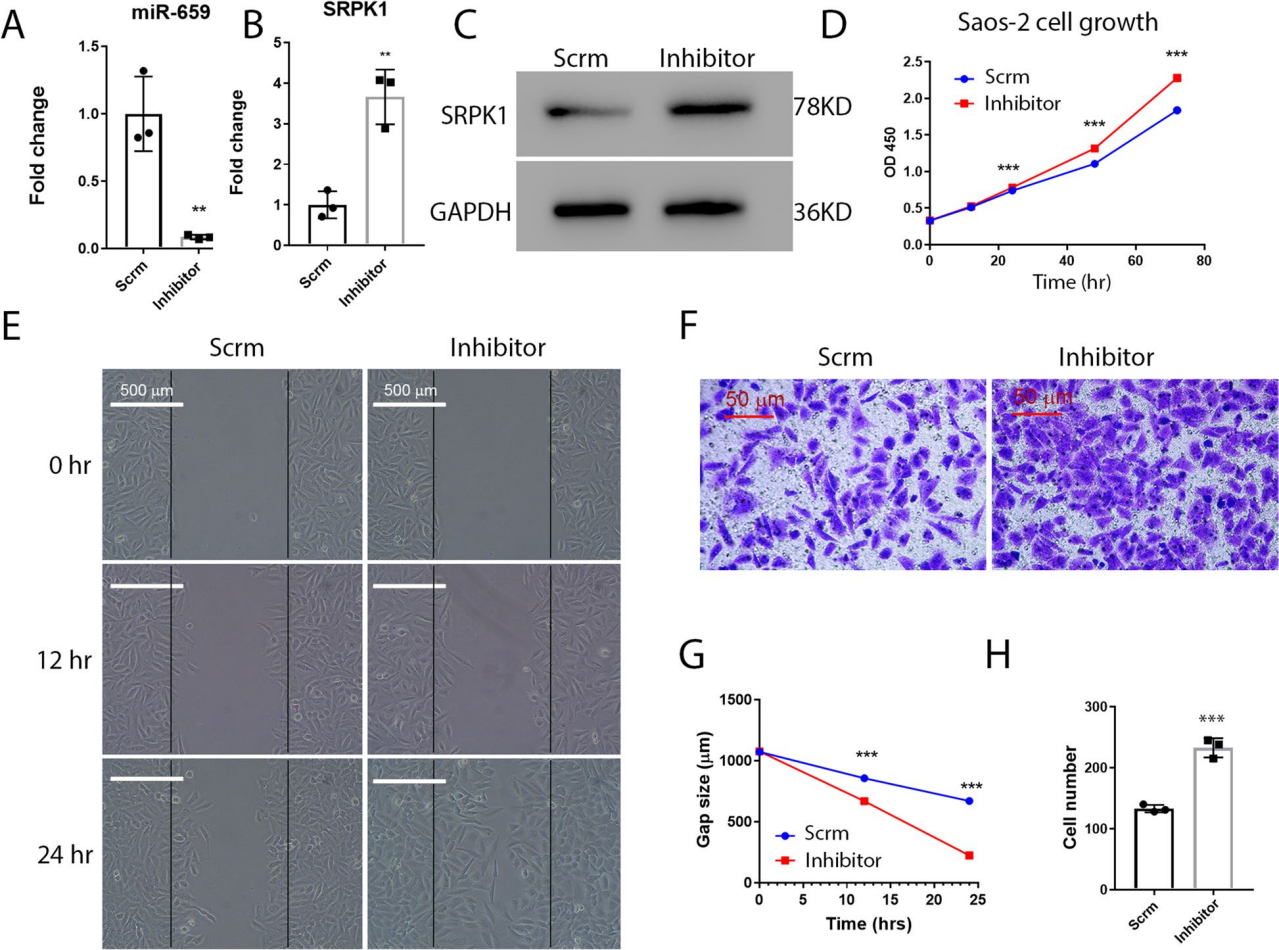
Inhibiting miR-659-3p increases osteosarcoma cells growth, migration, and invasion. **A** Transfection of miR-659-3p inhibitor decreased miR-659-3p level in Saos-2 osteosarcoma cells. **B** Real-time PCR revealed that inhibiting miR-659-3p increased *SRPK*1 mRNA expression by 3.7 folds in Saos-2 cells. Transfection of Has-miR-1225-5P inhibitor increase U2OS cells growth. **C** Western blot confirmed that SRPK1 expression was up-regulated on protein level by inhibiting miR-659-3p. SRPK1 and GAPDH western blots were performed on different gels. **D** Inhibiting miR-659-3p promoted Saos-2 cell growth. **E** Representative pictures of Saos-2 cells wound gap at 0, 12, and 24 h of culture. **F** Representative pictures of crystal violet stained Saos-2 cells invaded through transwells to the bottom chamber of the wells. **G** Quantification of Saos-2 cells wound gap after 12 and 24 h (*n* = 3), which showed miR-659-3p inhibitor significantly increased wound healing in cell culture dishes. H. Quantification of Saos-2 cells in the bottom chambers (*n* = 3). ** indicates *P* < 0.01 and *** indicates *P* < 0.001 compared to scramble control

The finding that miR-659-3p mimic inhibits *SRPK*1 expression and inhibits osteosarcoma cells proliferation and migration, leads us to further hypothesis that inhibiting miR-659-3p in osteosarcoma cells will promote cell proliferation and migration. To test this hypothesis, we transfected Saos-2 osteosarcoma cells with miR-659-3p inhibitor. Quantitative real-time PCR confirmed that miR-659-3p expression was significantly downregulated by miR-659-3p inhibitor transfection (Fig. 3A). Real-time PCR also revealed that *SRPK*1 mRNA expression was upregulated by inhibiting miR-659-3p (Fig. 3B). Further analysis via Western blot confirmed SRPK1 protein expression was also upregulated (Fig. 3C). After scrambled miRNA or miR-659-3p inhibitor transfection, we assayed cell proliferation. Figure 3 D showed that inhibiting miR-659-3p significantly increased Saos-2 osteosarcoma cells proliferation. By inhibiting miR-659-3p, there is a significantly higher migration capability, as indicated by the wound healing experiment (Fig. 3 E, G). Furthermore, the transwell invasion assay demonstrated that inhibiting miR-659-3p significantly increased Saos-2 osteosarcoma cells invasion as indicated by the increased number of cells found in the lower chamber of the transwells (Fig. 3F, H). Ultimately, this data demonstrated that miR-659-3p negatively regulates *SRPK*1 expression and plays a role in osteosarcoma cells proliferation, migration, and invasion.

### MiR-659-3p regulates osteosarcoma cells proliferation by affecting G1/G0 phase exit

**Fig. 4:**
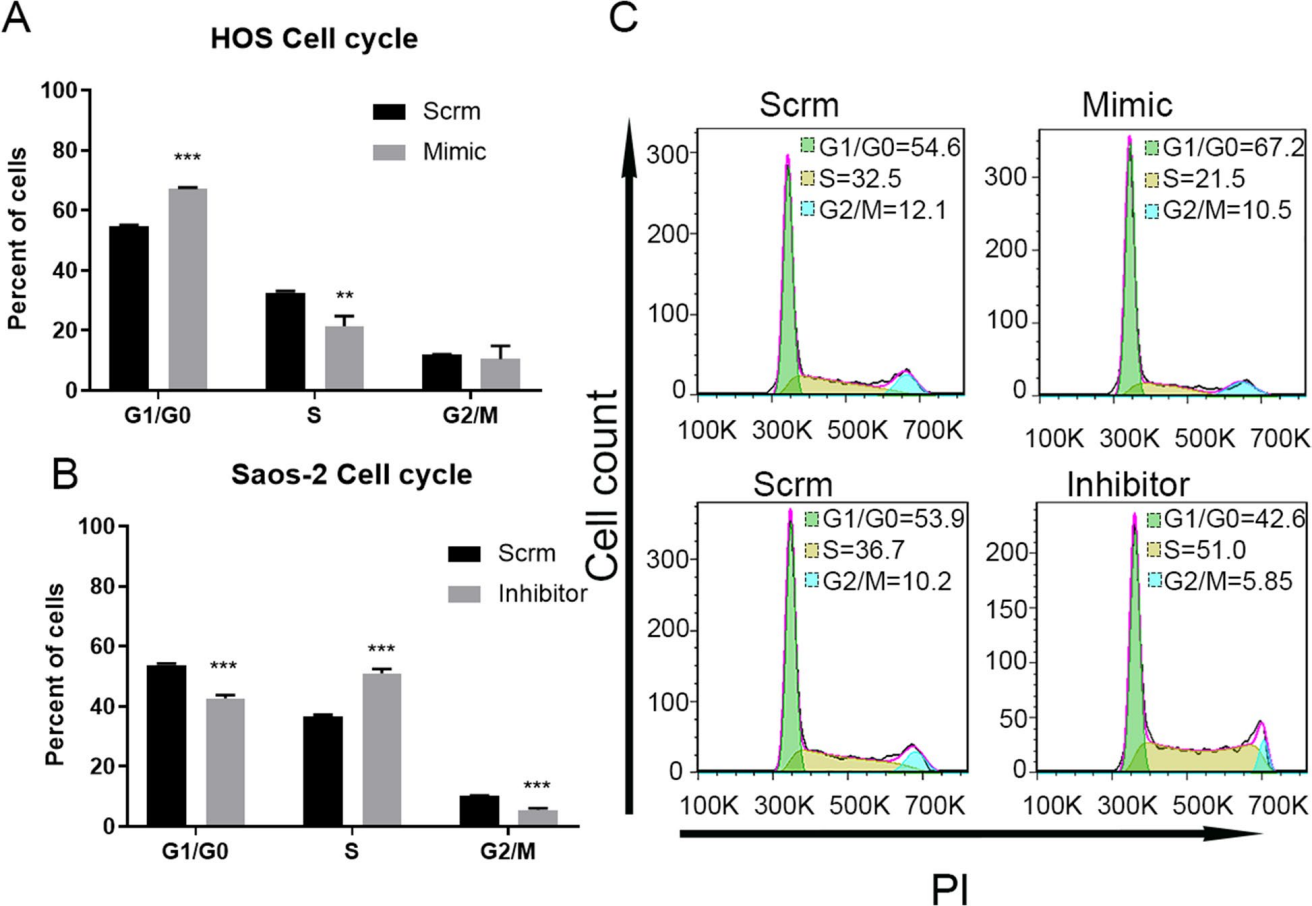
MiR-659-3p inhibits osteosarcoma cells cell cycle from G1/G0 to S phase transition. **A** Transfection of miR-659-3p mimic in HOS osteosarcoma cells, increased G1/G0 phase cells from 54.7% to 67.1%. **B** Transfection of miR-659-3p inhibitor in Saos-2 cells decreased G1/G0 phase cells from 53.8% to 42.7%. C. Representative FACS plot showed the increase and decrease percentage of G1/G0 phase cells after transfection with miR-659-3p mimic or inhibitor compared to scramble control. ** indicates *P* < 0.01 and *** indicates *P* < 0.001 compared to scramble control

SRPK1 was first characterized as a cell cycle regulated protein [39]. The findings that miR-659-3p targeted *SRPK*1 3’UTR, negatively regulated *SRPK*1 expression, and regulated osteosarcoma cell proliferation lead us to further explore the hypothesis that miR-659-3p affects osteosarcoma cell cycle progression through *SRPK*1. To test this hypothesis, we performed cell cycle profiling of osteosarcoma cells by FACS analysis. The HOS or Saos-2 cells were transfected with miR-659-3p mimic or inhibitor, respectively. After the cells were fixed, permeabilized, and stained with propidium iodide (PI), the cells were analyzed by FACS analysis. As shown in Fig. 4 A and C, transfection of miR-659-3p mimic increased the percentage of G1/G0 cells from 54.66% ± 0.50 (scramble control) to 67.09% ± 0.57 (*P* < 0.001). Inversely, inhibiting miR-659-3p by transfection of miR-659-3p inhibitor significantly reduced the percentage of Saos-2 osteosarcoma cells (from 53.79% ± 0.49 to 42.71% ± 1.09, *P* < 0.001, Fig. 4 B and C). This data indicated that miR-659-3p affects osteosarcoma cells' G1/G0 phase exit by negatively regulating *SRPK*1 expression.

### Overexpression of *SRPK*1 attenuates inhibition effect of miR-659-3p in osteosarcoma cells proliferation, migration and cell cycle progression

**Fig. 5:**
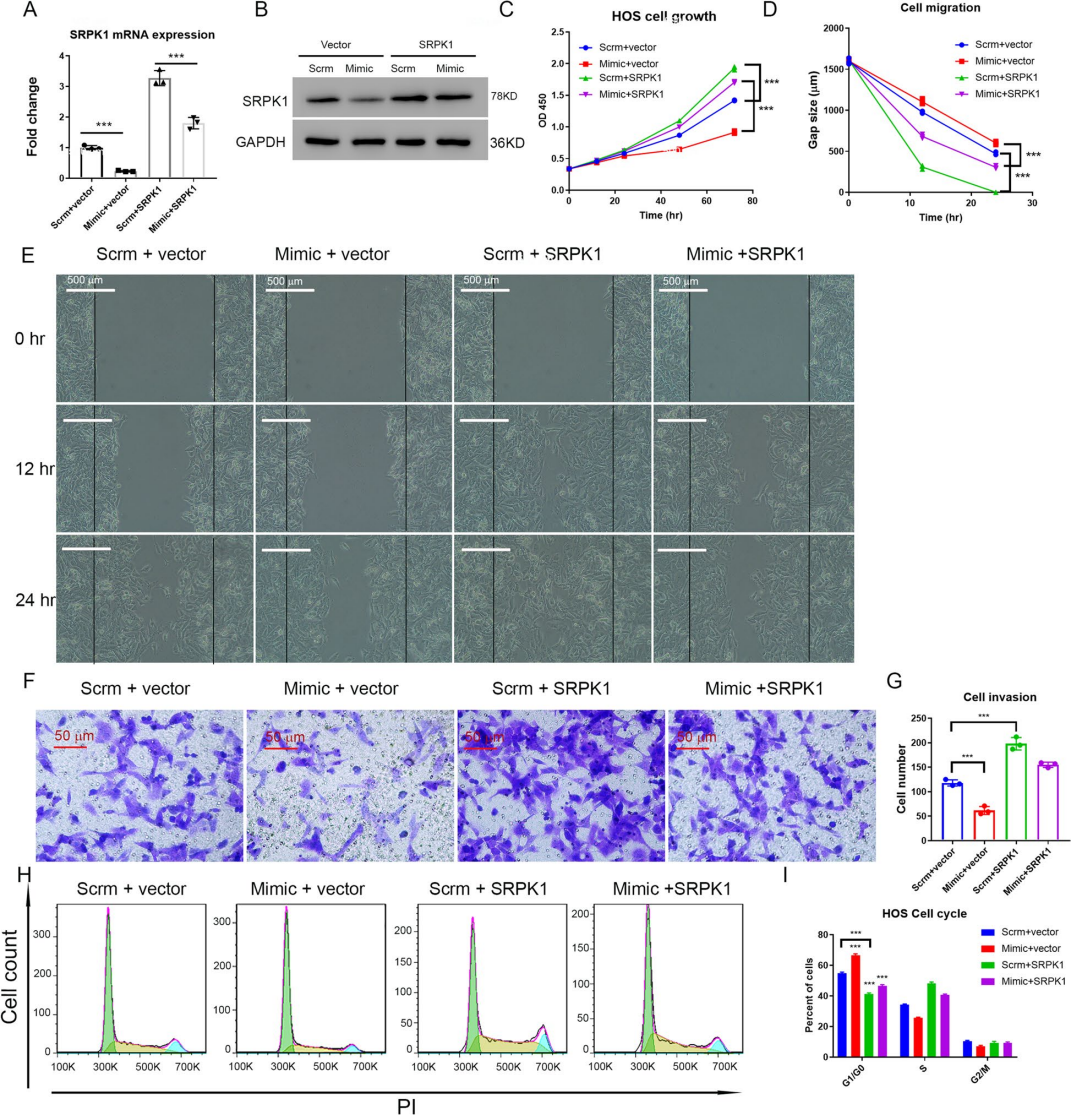
Overexpression of *SRPK*1 attenuated inhibition of miR-659-3p in osteosarcoma cells growth, migration, and invasion. **A** Real-time PCR showed that overexpression of *SRPK*1 restored *SRPK*1 mRNA expression in miR-659-3p mimic transfected HOS cells. **B** Western blot confirmed that overexpression of *SRPK*1 restored SRPK1 protein level in HOS osteosarcoma cells (lane 4). *SRPK*1 expression was knocked down by miR-659-3p mimic (lane 2). SRPK1 and GAPDH western blots were performed on different gels. **C** Overexpression of *SRPK*1 increased HOS cells growth (Green line and Blue line, *P* < 0.001 *n* = 3), and attenuated miR-659-3p inhibition of HOS cells growth (Purple line and Red line, *P* < 0.001 *n* = 3). **D** Quantification of HOS cells wound gap after 12 and 24 h culture. **E** Representative pictures of HOS cells wound gap after 12 and 24 h culture showed that overexpression of *SRPK*1 accelerated wound healing in the dishes. **F** Transwell invasion assay showed that inhibition of *SRPK*1 expression by miR-659-3p mimic decreased HOS cells invasion and overexpression of *SRPK*1 increased HOS cells invasion. **G** Quantification of cell numbers in the lower chambers of transwells. **H** Representative FACS histogram plots showed that *SRPK*1 overexpression decreased G1/G0 phase arrest induced by miR-659-3p. **I** The average percentage of each phase in cell cycle (*n* = 3 each group) showed that *SRPK*1 overexpression decreased G1/G0 phase arrest induced by miR-659-3p. *** indicates *P* < 0.001 in the indicated comparison

To further prove that miR-659-3p regulates osteosarcoma cells proliferation, migration, and invasion through *SRPK*1, we utilized the *SRPK*1 gain-of-function strategy. HOS osteosarcoma cells were co-transfected with pCDNA3.1 expression vector containing human *SRPK*1 cDNA sequence (*SRPK*1/pCDNA) and miR-659-3p mimic. Combinations of empty pCDNA3.1 vector with scramble miRNA, *SRPK*1/pCDNA with scramble miRNA, and empty pCDNA3.1 vector with miR-659-3p were used as controls. The expression of *SRPK*1 on mRNA level and protein level were confirmed by quantitative real-time PCR (Fig. 5A) and Western blot (Fig. 5B). As expected, empty pCDNA3.1 vector with miR-659-3p mimics transfected cells expressed reduced *SRPK*1 mRNA and protein, which further confirmed that miR-659-3p targets *SRPK*1 expression and *SRPK*1/pCDNA with scramble miRNA transfected cells increased *SRPK*1 expression (Fig. 5A, B). Overexpression of *SRPK*1 significantly increased HOS osteosarcoma cells proliferation (Fig. 5C, compare green line with blue line) and rescued miR-659-3p inhibition of HOS cells proliferation (Fig. 5C, compare purple line with red line). In the wound healing assay, the gap sizes of *SRPK*1 overexpressed cells were significantly smaller than the controls (Fig. 5D and E, compare green line with blue line, and purple line with red line), which indicated that overexpression of *SRPK*1 increased osteosarcoma cells migration. In contrast, knocking down *SRPK*1 expression by miR-659-3p mimics decreased osteosarcoma cells migration (Mimic + vector) (Fig. 5D and E). However, *SRPK*1 overexpression mitigated inhibition of osteosarcoma cells migration by miR-659-3p (Fig. 5D and E, compare purple line with red line). To further elucidate the role of *SRPK*1 in miR-659-3p inhibition of osteosarcoma cells invasion, we used the same groups of cells as above and performed a transwell cell invasion assay. This experiment confirmed that miR-659-3p reduced osteosarcoma cell invasion (Fig. 5F and G). Moreover, *SRPK*1 overexpression increased the capability of osteosarcoma cell invasion and rescued the inhibitory effect of miR-659-3p (Fig. 5F and G). Thus, we further investigated whether *SRPK*1 overexpression can rescue the effect of miR-659-3p on osteosarcoma cell G1/G0 phase exit. The *SRPK*1 overexpressed cells exhibited significantly lower G1/G0 cell percentage compared to the controls (Fig. 5H and I). When *SRPK*1 was overexpressed in scramble miRNA transfected cells, the percentage of G1/G0 cells reduced from 54.76% ± 0.82 (scramble with empty vector control) to 41.18% ± 0.81 (*P* < 0.0001). Consistent with the data in Fig. 4, miR-659-3p increased G1/G0 cells (66.60% ± 0.88) compared to control (*P* < 0.0001). However, overexpression of *SRPK*1 significantly reduced the percentage of G1/G0 cells (46.60% ± 0.77, vs. 66.60% ± 0.88, *P* < 0.0001). This data indicated that inhibition of cell proliferation, migration, invasion, and cell cycle progression by miR-659-3p was at least in part through targeting *SRPK*1.

### MiR-659-3p inhibits osteosarcoma cell growth by promoting cell apoptosis

**Fig. 6:**
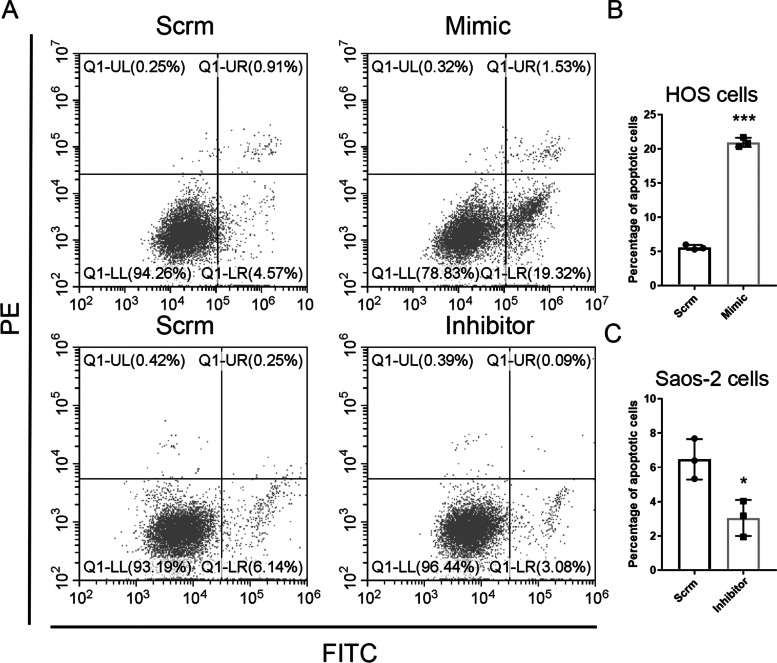
Mir-659-3p promotes cell apoptosis in osteosarcoma cells. **A** Representative FACS plots of osteosarcoma cells transfected with either control miRNA or miR-659-3p mimic (upper panels), or miR-659-3p inhibitor (lower panels). **B** Average percentage of apoptotic cells in HOS cells transfected with miR-659-3p mimic compared with control miRNA (*n* = 3 *P* < 0.001). **C** Average percentage of apoptotic cells in Saos-2 cells transfected with miR-659-3p inhibitor compared with control miRNA (*n* = 3 *P* < 0.05)

*SRPK*1 plays a critical role in cancer bypassing apoptosis in several cancer types, such as colorectal cancer [40], breast cancer [14]. We thus hypothesized that the mechanism of miR-659-3p inhibiting osteosarcoma cell growth at least in part is that miR-659-3p down regulates SRPK1 expression and promotes osteosarcoma cell apoptosis. To test this hypothesis, we transfected HOS cells and Saos-2 cells with miR-659-3p mimic and inhibitor respectively. Apoptosis of the cells were analyzed by Annixin V binding and PI staining. Double negative staining indicates the live cells, Annixin V single positive indicates the cells are in the early apoptotic stage and Annixin V/PI double positive indicates the cells are in the late stage of apoptosis. As shown in upper panels of Fig. 6A and B, overexpression of miR-659-3p in HOS osteosarcoma cells increased percentage of apoptotic cells from 5.59 ± 0.36% to 20.95 ± 0.68% (*P* < 0.0001). Inversely, inhibiting miR-659-3p in Saos-2 cells by transfection miR-659-3p inhibitor decreased percentage of apoptotic cells from 6.47 ± 1.18% to 3.05 ± 1.05% (*P* < 0.05, lower panels Fig. 6A and C)). This data indicated that miR-659-3p promoted cell apoptosis in osteosarcoma cells.

### MiR-659-3p inhibits osteosarcoma tumor growth and metastasis in mouse model

**Fig. 7:**
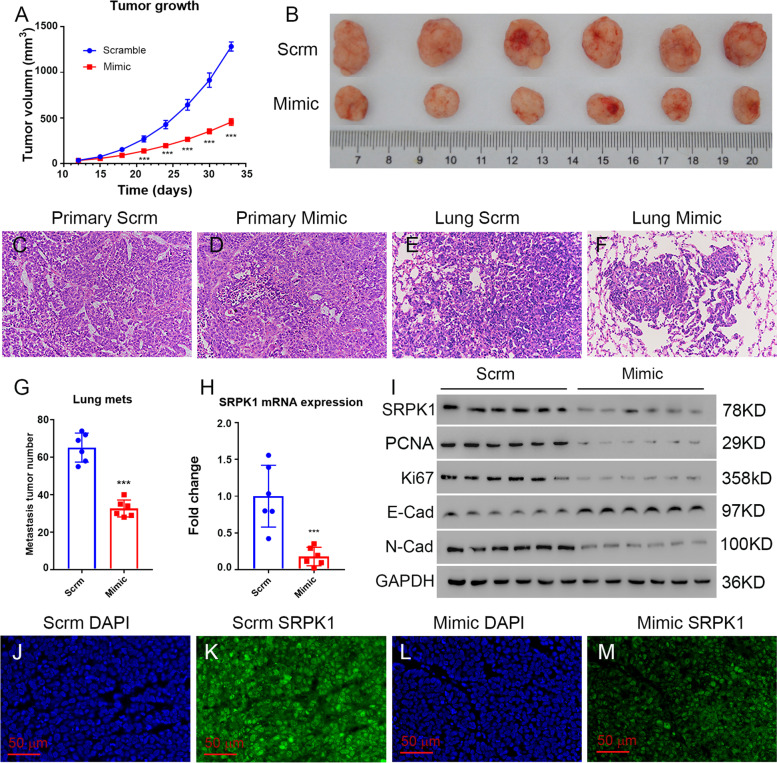
MiR-659-3p inhibits osteosarcoma tumor growth and metastasis *in vivo*. **A** Tumor growth curve in nude mice indicated that miR-659-3p inhibited tumor growth (red line) compared to scramble miRNA treatment (blue line) (*n* = 6, *P* < 0.001). **B** Picture of tumors at the end time point showed smaller sizes of miR-659-3p treated tumors compared to scramble miRNA treated. **C** Representative histology of primary tumor from scramble miRNA injected mouse. **D** Representative histology of primary tumor from miR-659-3p mimic injected mouse. **E** Representative histology of lung metastasis tumor from scramble miRNA injected mouse. **F** Representative histology of lung metastasis tumor from miR-659-3p mimic injected mouse. **G** Quantification of lung metastasis from the lungs (*n* = 6, *P* < 0.001). H. Real-Time PCR showed *SRPK*1 expression on mRNA level was downregulated in miR-659-3p mimic treated tumors compared to scramble miRNA treated tumors (*n* = 6). **I** Western blot showed protein expression in the tumor tissue treated with miR-659-3p mimic compared to scramble miRNA treated. All western blots were performed on different gels. **J** Representative image of DAPI staining of primary tumor from miR-659-3p mimic injected mouse. **K** Representative image of SRPK1 immunostaining of primary tumor from scramble miRNA injected mouse. **L** Representative image of DAPI staining of primary tumor from scramble miRNA injected mouse. **M** Representative image of SRPK1 immunostaining of primary tumor from miR-659-3p mimic injected mouse. *** indicates *P* < 0.001 in the indicated comparison

The findings above lead us to ask further whether miR-659-3p can inhibit tumor growth *in vivo*. A subcutaneous tumor mouse model was established by injecting HOS osteosarcoma cells into the nude mice. The mice were intravenously injected with miR-659-3p or scramble miRNA a week after osteosarcoma cells injection. After the tumors were visible, the tumor sizes were measured every 3 days and the tumor growth curves were plotted (Fig. 7A). Administration of miR-659-3p significantly reduced tumor growth *in vivo* compared with the scramble miRNA control (Fig. 7A). At the endpoint of the experiment, the tumors dissected from the miR-659-3p mimic injected mice exhibited smaller sizes compared with the mice injected with control miRNA (Fig. 7B). Histology analysis of the tumor tissues confirmed they were osteosarcoma (Fig. 7C, D). MiR-659-3p inhibited osteosarcoma cells migration and invasion *in vitro* (Fig. 2), which led us to further investigate whether miR-659-3p inhibits osteosarcoma metastasis *in vivo*. At the endpoint of the *in vivo* experiment, the whole lungs were dissected from all the mice. Metastatic tumors were counted from 5 interval sections of the lungs. Interestingly, the mice injected with miR-659-3p mimic had a significantly lower number of metastatic tumors compared with control miRNA injected mice (Fig. 7E-G). The sizes of the metastatic tumors from miR-659-3p injected mice were smaller compared with that from control miRNA injected mice visualized by H&E staining (Fig. 7E, F). Furthermore, real-time PCR and Western blot assays revealed that tumors from mice that were injected with miR-659-3p mimic had lower levels of *SRPK*1 mRNA and protein than those injected with control miRNA (Fig. 7 H and I). Immunofluorescent staining further verified that the protein expression of SRPK1 was downregulated by miR-659-3p (Fig. 7 L, M) compared with scramble miRNA (Fig. 7J, K). These findings indicated that miR-659-3p reduced *SRPK*1 expression both at mRNA and protein levels *in vivo*. This data suggested that miR-659-3p not only inhibited osteosarcoma growth but also inhibited lung metastasis *in vivo* by inhibiting *SRPK*1 expression in osteosarcoma cells. To further investigate the possible underline mechanisms of miR-659-3p/SRPK1, we performed protein level analysis of proliferative markers and epithelial-to-mesenchymal transition (EMT) markers. Interestingly, the primary tumors from miR-659-3p injected mice expressed less proliferative markers PCNA and Ki67 (Fig. 7I), indicating the tumor cells were less proliferative *in vivo*. Upregulation of E-Cadherin and downregulation of N-Cadherin is an indication of suppression of EMT [41]. We found that the expression of E-Cadherin in the primary tumors was upregulated, and the expression of N-Cadherin was downregulated (Fig. 7I), which indicated that miR-659-3p may inhibit osteosarcoma EMT.

## Discussion

One of the major reasons that patients with metastasis or relapse experience with poor prognosis and that there is low survival rate is due to a lack of efficient treatment options for metastatic osteosarcoma [2, 42]. However, the mechanisms of osteosarcoma malignancy and metastasis are not fully understood. Abnormal miRNA expression may play a role in osteosarcoma malignancy transformation and metastasis [27, 28, 43]. In this study, we found a novel miR-659-3p-*SRPK*1 axis that regulates osteosarcoma cells growth, migration, invasion in vitro*,* and tumor progression and metastasis *in vivo*. MiR-659-3p was found significantly down-regulated in osteosarcoma compared to normal bone cells. Although the clinical dataset is under power, the trend clearly showed that higher miR-659-3p expression patients had better prognosis. Therefore, it is possible that the use of miR-659-3p alone or in combination with other miRNAs as an osteosarcoma biomarker for treatment strategies.

As previously mentioned, SRPK1 plays a role in many other cancers, and thus the mechanisms of the action of SRPK1 in some cancers were also explored. SRPK1 interacts with several oncogenic signaling pathways, such as Akt/eIF4E/HIF-1/VEGF signaling pathway [44, 45], Erk/MAPK signaling pathway [46, 47], PI3K/AKT/mTOR signaling pathway [48], TGF-β signaling pathways [49], and Wnt/β-catenin signaling pathway [50]. In this study, we revealed that SRPK1 may regulate cell proliferation molecules such as PCNA and Ki67 to play a critical role in osteosarcoma cell proliferation and tumor progression. However, the detailed mechanisms of how SRPK1 regulates PCNA and Ki67 expression warrant further investigation and will be reported in a separate manuscript.

The present study hinted at least two different mechanisms of *SRPK*1 in osteosarcoma. First, *SRPK*1 plays a role in the osteosarcoma cell cycle regulation. Our current data indicated that inhibiting *SRPK*1 by miR-659-3p increased osteosarcoma cells G1/G0 phase arrest (Fig. 4 and 5). A similar function of *SRPK*1 has been reported in gastric cancer, *SRPK*1 knocking down increased G1/G0 phase arrest in gastric cancer cells [10]. However, which downstream molecules are playing a role in G1/G0 phase arrest need further investigation. Second, cancer cells can bypass apoptosis through a series of complex mechanisms. *SRPK*1 plays a critical role in cancer bypassing apoptosis in several cancer types, such as colorectal cancer [40], breast cancer [14]. Therefore one of the explanations miR-659-3p inhibits osteosarcoma cells growth, migration and invasion, and osteosarcoma tumor progression is that miR-659-3p increases osteosarcoma cell apoptosis by downregulating SRPK1 expression and further downregulated SRPK1 downstream targets such as PCNA and Ki67. Indeed, our apoptosis assay showed that miR-659-3p mimic promoted cell apoptosis in HOS osteosarcoma cells and inhibiting miR-659-3p inhibited cell apoptosis in Saos-2 osteosarcoma cells (Fig. 6).

Currently, metastasis is the major hurdle for osteosarcoma treatment [2]. EMT plays a crucial role in tumorigenesis and enhances metastasis of many cancer types [41]. Therefore, targeting EMT is of great interest in counteracting osteosarcoma metastasis. In this study, we found that injecting miR-659-3p *in vivo* reduced N-cadherin expression and increased E-cadherin expression in the mouse osteosarcoma model, which indicated that miR-659-3p inhibited EMT of osteosarcoma cells. However, the detailed molecular mechanisms underlining the inhibition of EMT are not clear, and we will further investigate the possible molecular mechanisms and will report them in a separate manuscript.

Taken together, this study revealed that miR-659-3p negatively regulates *SRPK*1 expression in osteosarcoma cells, inhibits osteosarcoma cells growth, migration, and invasion *in vitro*. To our best knowledge, this is the first time it revealed that miR-659-3p plays a role in osteosarcoma. Moreover, our *in vivo* mouse model further proved that miR-659-3p inhibits tumor growth and metastasis. Inhibiting *SRPK*1 has been proved to have an antitumor effect in other cancer types, such as leukemia [51] and melanoma [52]. *SRPK*1 inhibitors have failed to have suitable absorption, circulation, digestion, exclusion, and noxiousness to test further in clinical trials [53]. This study provided evidence not only that *SRPK*1 could be used as a therapeutic target for osteosarcoma treatment, but also that miR-659-3p could potentially be used as a tool for targeting *SRPK*1 in osteosarcoma treatment.

## Supplementary Information

**Additional file 1.** Original files of Western blots.

**Additional file 2: Supplemental Fig. 1. **Overlapping gene numbers between TargetScan results and Differential Expressed miRNAs (DEMs).

**Additional file 3:** **Supplemental file 1.**

**Additional file 4:** **Supplemental file 2.** 

## Data Availability

The data generated and/or analyzed in this study are included in this article and supplemental information files. Dr. Yubao Gong is the contact person for requesting data of this study (gongyb@jlu.edu.cn).
