# Supplementary material for: MiR-659-3p inhibits osteosarcoma progression and metastasis by inhibiting cell proliferation and invasion via targeting SRPK1
Source: BMC Cancer. 2022 Aug 29;22:934. doi: 10.1186/s12885-022-10029-0 (PMC9425973; doi:10.1186/s12885-022-10029-0)

Original files of Western blots

(Western blots with red boxes are the ones used in the manuscript figures)

Fig. 1G: Replicate 1

SRPK1

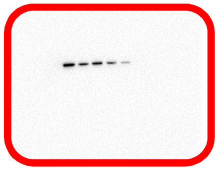

GAPDH

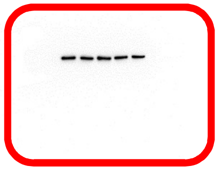

Replicate 2

SRPK1

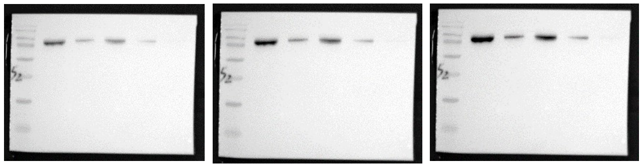

GAPDH

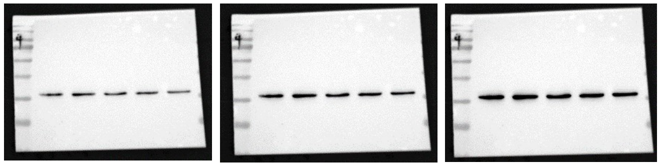

Replicate 3

SRPK1

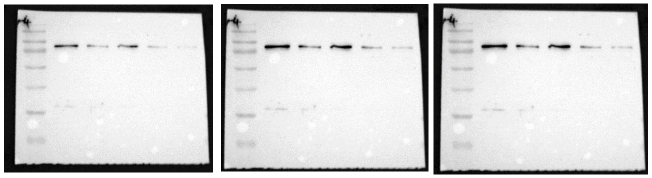

GAPDH

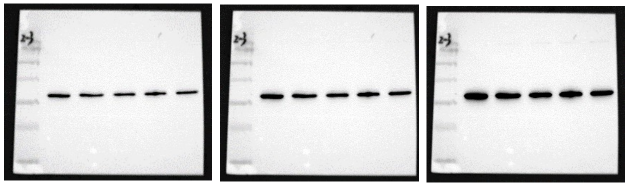

Fig. 2C: Replicate 1

SRPK1

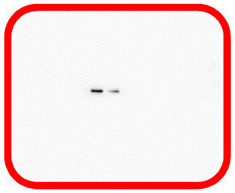

GAPDH

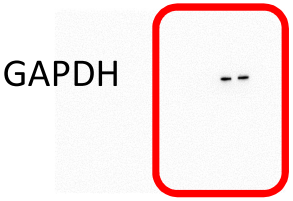

Replicate 2

SRPK1

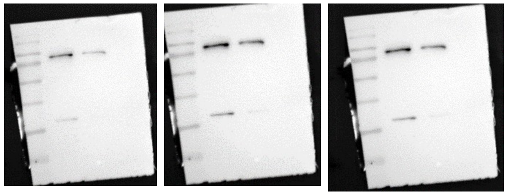

GAPDH

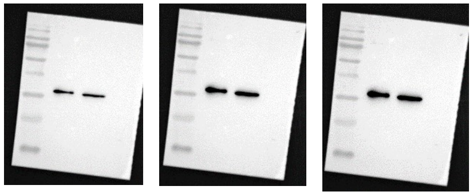

Replicate 3

SRPK1

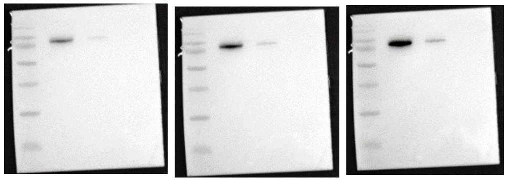

GAPDH

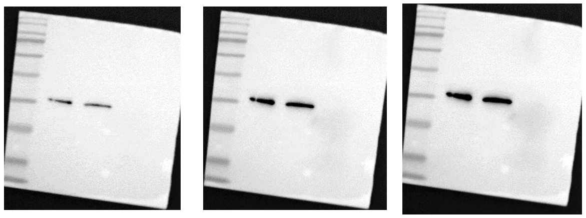

Fig. 3C:

Replicate 1

SRPK1

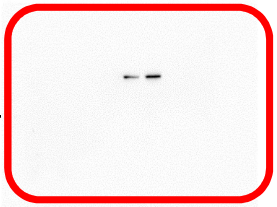

GAPDH

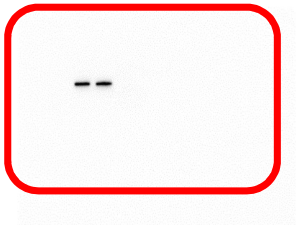

Replicate 2

SRPK1

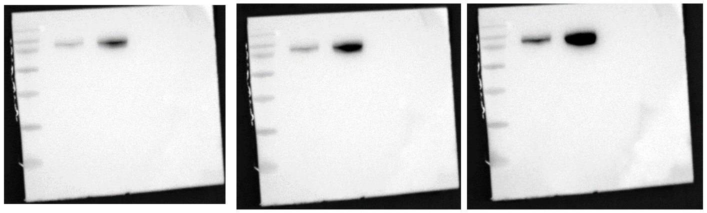

GAPDH

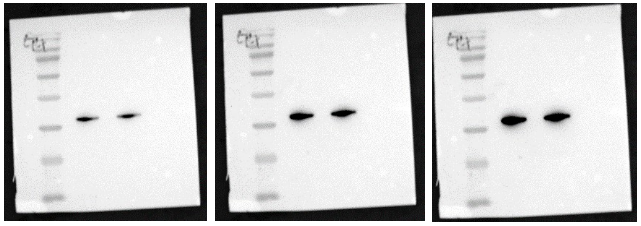

Replicate 3

SRPK1

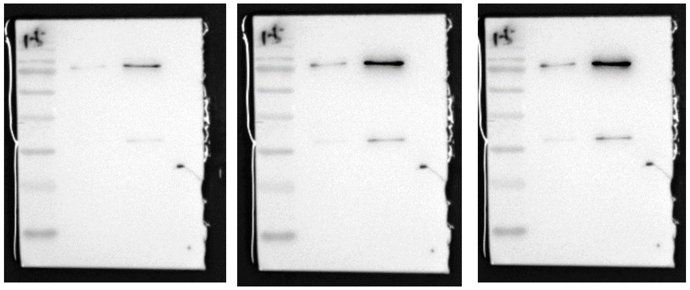

GAPDH

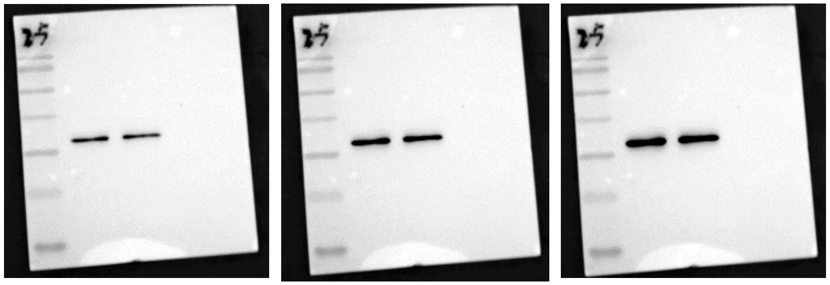

Fig. 3C:

Replicate 3

SRPK1

GAPDH

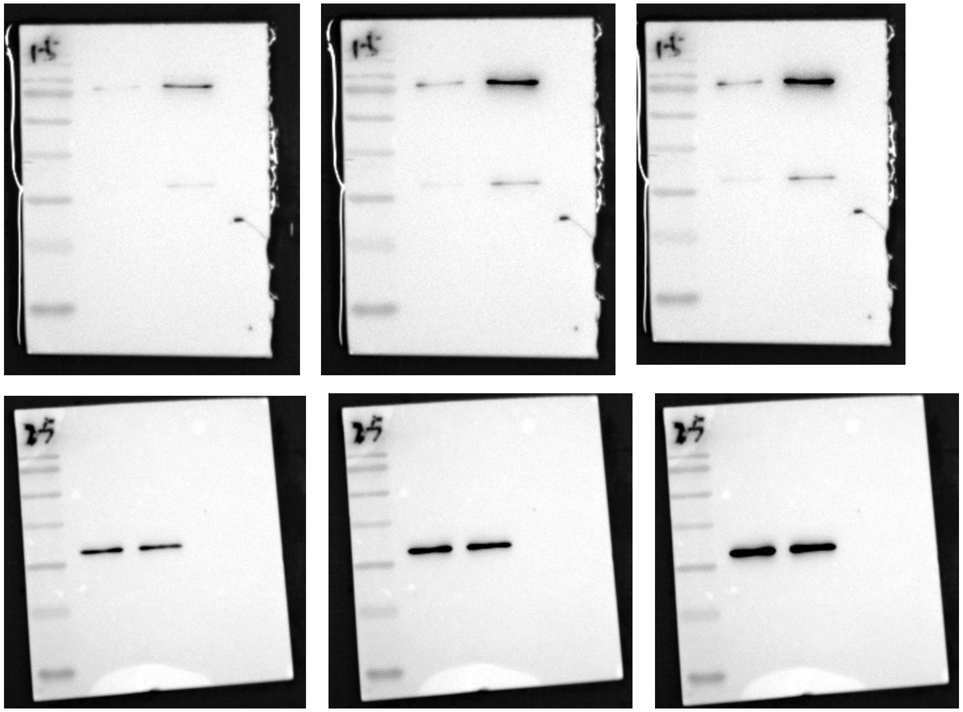

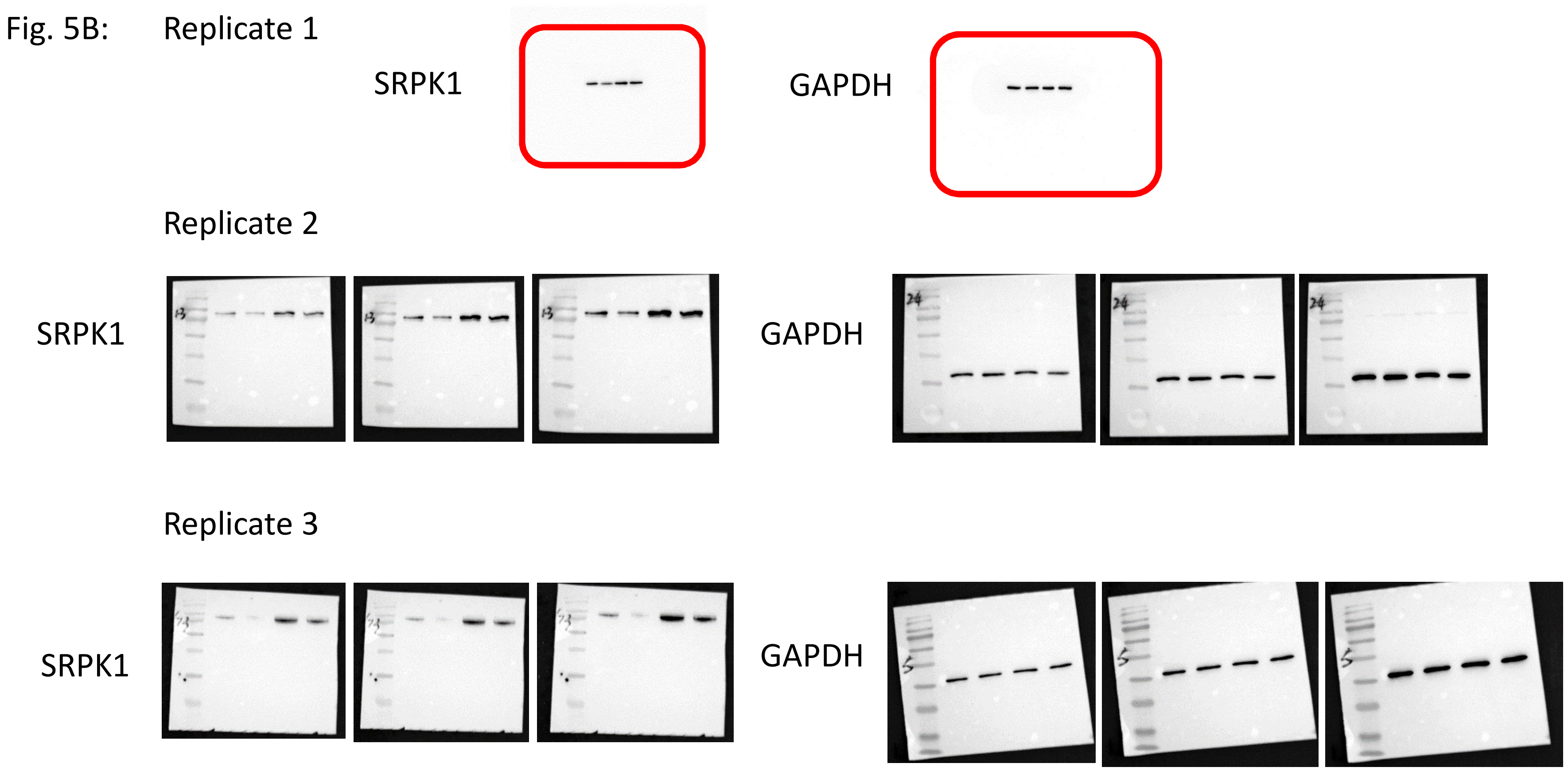
Fig. 5B:
Replicate 1
SRPK1
GAPDH
Replicate 2
SRPK1
GAPDH
Replicate 3
SRPK1
GAPDH

Fig. 7I:

Replicate 1

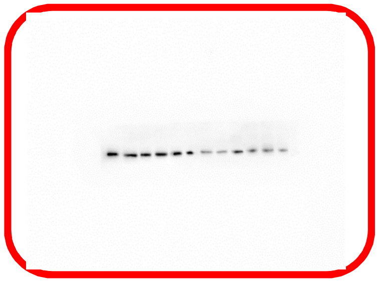

SRPK1

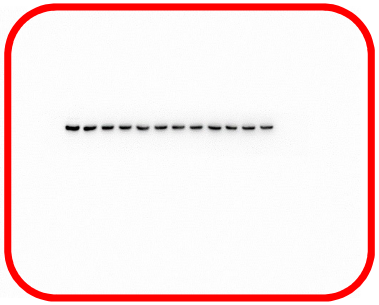

GAPDH

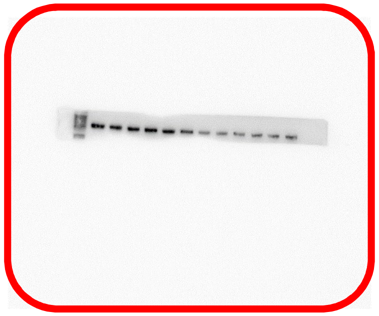

Ki67

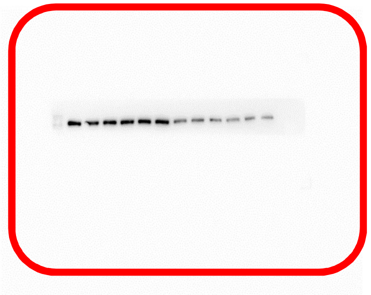

N-Cad

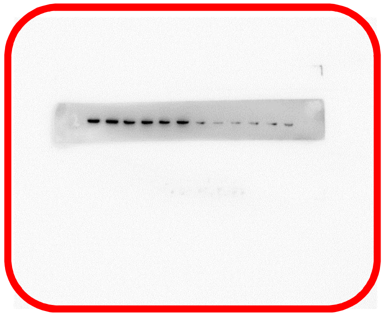

PCNA

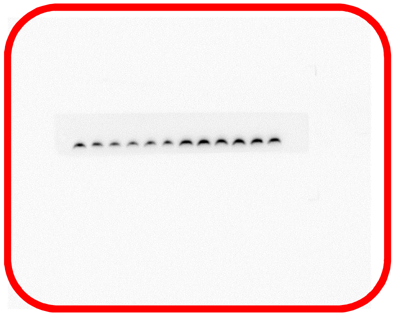

E-Cad

Fig. 7I: Replicate 2 (with multiple exposure)

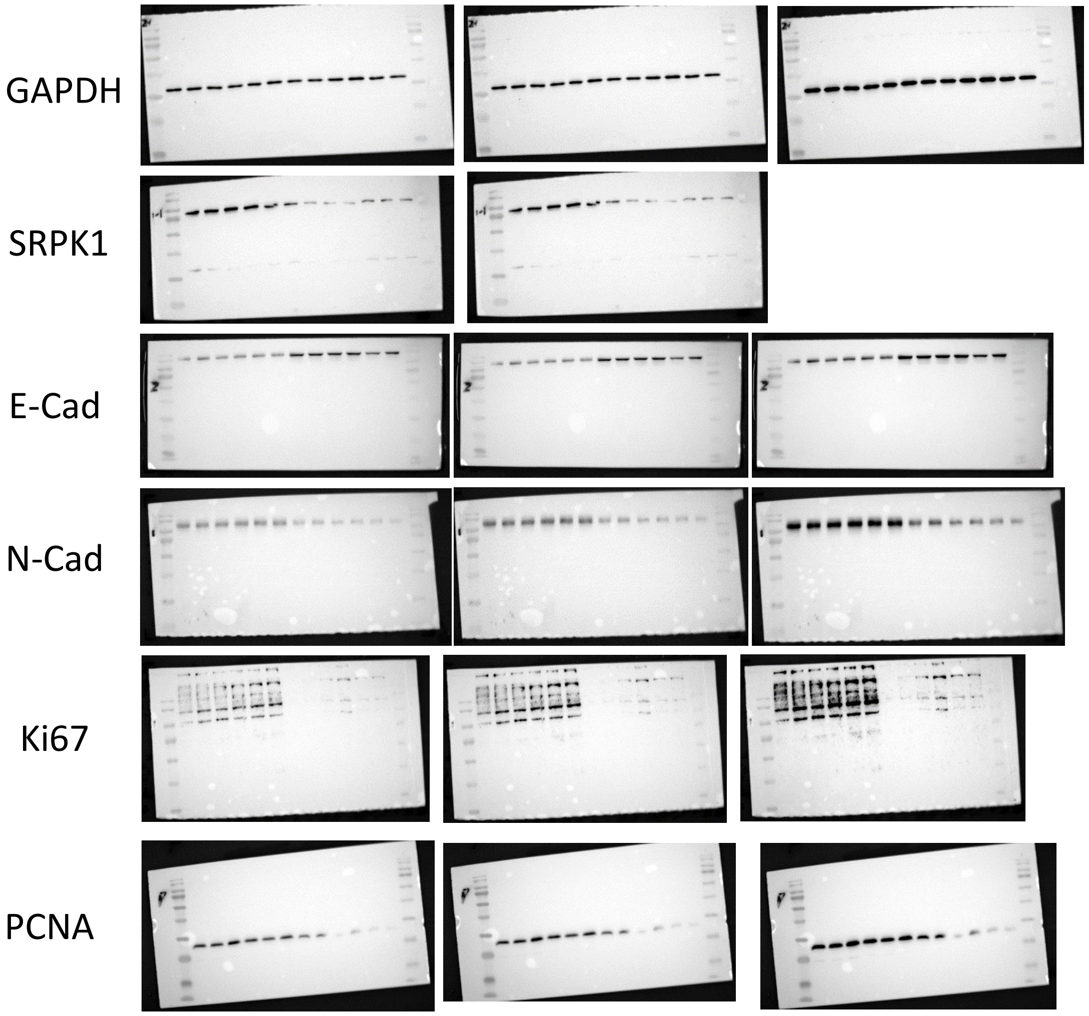

Fig. 7I: Replicate 3 (with multiple exposure)

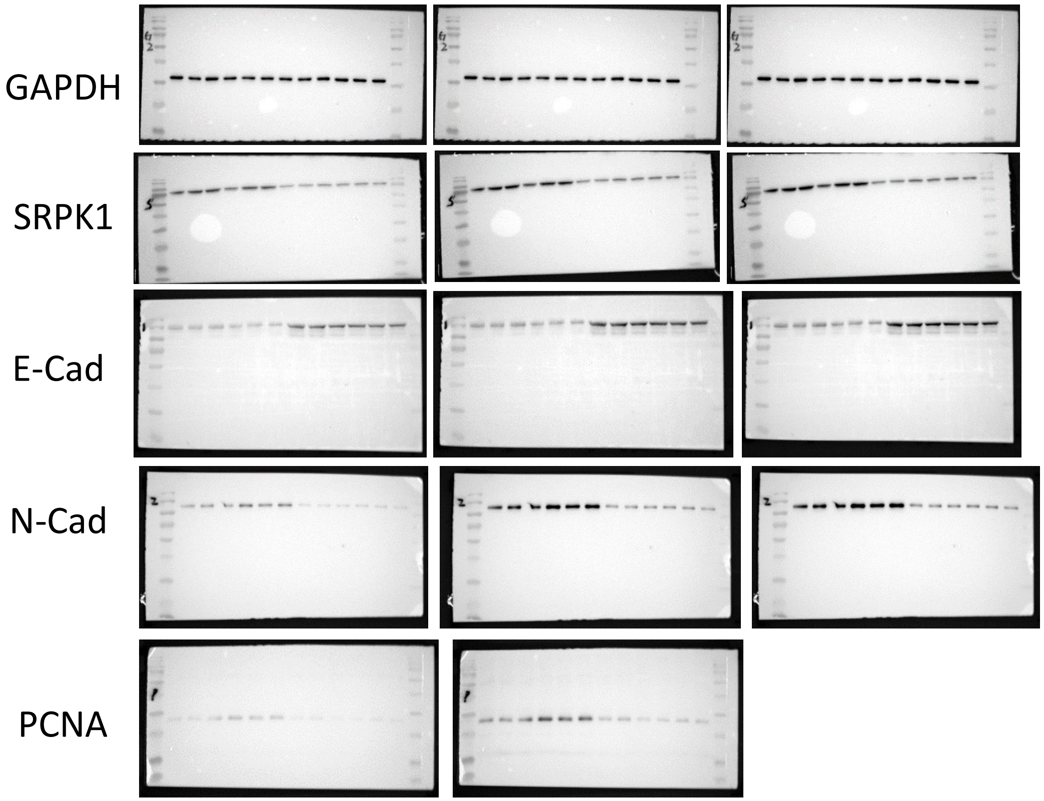

Supplement: Supplementary file 1 — Additional file 1. Original files of Western blots. [file 12885_2022_10029_MOESM1_ESM.pdf]
