# Supplementary material for: MiR-659-3p inhibits osteosarcoma progression and metastasis by inhibiting cell proliferation and invasion via targeting SRPK1
Source: BMC Cancer. 2022 Aug 29;22:934. doi: 10.1186/s12885-022-10029-0 (PMC9425973; doi:10.1186/s12885-022-10029-0)

Supremental Fig.1 Overlapping gene numbers between TargetScan results and Differential Expressed miRNAs (DEMs)

Input files:

List names	number of elements	number of unique elements
DEMs	139	139
TargetScan	830	668
Overall number of unique elements		796

Image result:

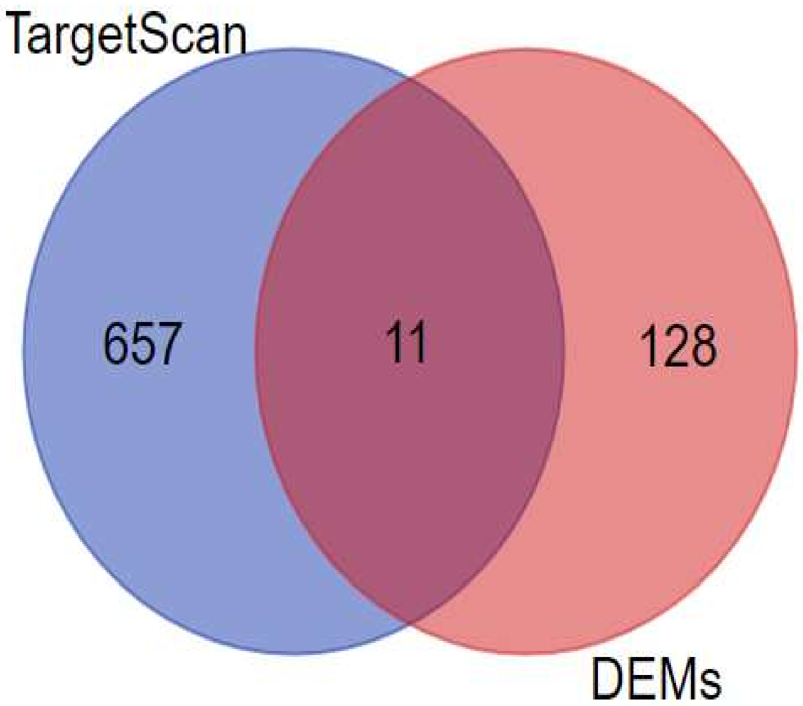

Supplement: Supplementary file 2 — Additional file 2: Supplemental Fig. 1. Overlapping gene numbers between TargetScan results and Differential Expressed miRNAs (DEMs). [file 12885_2022_10029_MOESM2_ESM.pdf]
