# Supplementary material for: MiR-659-3p inhibits osteosarcoma progression and metastasis by inhibiting cell proliferation and invasion via targeting SRPK1
Source: BMC Cancer. 2022 Aug 29;22:934. doi: 10.1186/s12885-022-10029-0 (PMC9425973; doi:10.1186/s12885-022-10029-0)
Supplement: Supplementary file 3 — Additional file 3: Supplemental file 1. [file 12885_2022_10029_MOESM3_ESM.docx]

Supplemental data

1. Wild Type SRPK1 gene 3’ UTR sequence (miR-659-3P targeting sequences are marked red)

GCCCCTGCCCAGCACCACAGCAGAGATCACACACTGACCCTCCGCCCTTCCCCTTCAAGCATTTTCCTCTTCCCTTTTCAGGGTGAAGCTCTTCCTTCAAGAGTTTCTAGATCTTGTTTTTTTTTTAATCCAACATGTTCATTTGGGTTTGCTTACTTGACCCTGTGGAGATCCCCACAGCCATTGGGCATCCTAGGTGAATTTGGCCTTGGTTGGGCTCTGCCAAAGACTAATGGACTAAAATGTGAAACAGCCTCTTGCCCTGTACCTTTCCTTCCCATTAGGACATCCTTTAAATTATAAGCATCCTTTTTGAAAAGAGCTATGAAGGTGTATGAGCCCATCCTTTTATTCATTGACTCTAAGAGTCAAATTTTCTAGTGCATATCCTATTGCCAGCATAAGGATGAGGAGGGGGAAAGGGTCTTAATTCTATGTACAGCAGAGACATTAAACTTGCTGTGTCCGGGCTGCATCATCTTCCTGGACTGTTTCTGTTGTTCTCTGTGTTCACATTTTTTCCTGCAACTTTTAAGCTACTGTCTTTTTTAAATAGCTATATGAACACCAAATTTGGGTACCATTTTATCACTGTTCAAAGCACTGTCAAATTCCTTTCATCCTTTAATAGTTAAGATCTTTGAATCTTCAGTCTGATTTTTAATGTAAGCAAAAACAGAACCATTGAATAGTAATTTCTTGAGAACCTCAGGTGTTCTATAAACAGTCCTTTCCTGTATGTCTTCTATTACCCTAAGACCAGAGTTATTTTGGTTGGTTGTTTTGTTTTATTTTTTGTTTTTGTATCCATGGCTGGCACTTTACTCATTGCACTTGAGTTTATTGCCCCATAACTAAAGGATCAGGATGATGGTAGAACGGAGATCTGGGTTTCAGAGCTTTCCCATTTAAGAAAAATAGATCTTGAGATTCTGATTCTTTTCCAAACAGTCCCCTGCTTTCATGTACAGCTTTTTCTTTACCTTACCCAAAATTCTGGCCTTGAAGCAGTTTTCCTCTATGGCTTTGCCTTTCTGATTTTCTCAGAGGCTCGAGTCTTTAATATAACCCCAAATGAAAGAACCAAGGGGAGGGGTGGGATGGCACTTTTTTTTGTTGGTCTTGTTTTGTTTTGTTTTTTGGTTGGTTGGTTCGTTATTTTTTAAGATTAGCCATTCTCTGCTGCTATTTCCCTACATAATGTCAATTTTTAACCATAATTTTGACATGATTGAGATGTACTTGAGGCTTTTTTGTTTTAATTGAGAAAAGACTTTGCAATTTTTTTTTTAGGATGAGCCTCTCCTAGACTTGACCTAGAATATTACATATTCCTCCAGTAAGTAATACTGAAGAGCAAAAGAGAGGCAGGATTGGGGTCACAGCCGCTTCTTCAGCATGGACCAAGTGGGCCTTGGGGATTGCAGCGTTCTCGAAGTGGCTGTAGGACTCGAATTTACAGAAAGCCACAGAGGTGCAACTTGAGGCTCTGCTAGCAAGCCACCAGTGAGGCTATTGGGTAACCACCTTTCTATACAGGAGATTGGAATCTACTTTGTCATTTATCCACCACAGTGACAAAGGAAAAGTGGTGCCGTTATGCAATCCATTTAACTCATAAACATATTACTCTGAGTAACTGGCCAGCCATTCATCGGATCCTTCATTGGGTACTCCTGAAATCAGACATGTTCCTGTAGAAAGAATTTTAAGTTAGGCTTTCTATGCACCTATCAAGAATCAAGAGAATAGATTGTATCAAACAACGGCAGGGAAATCCTTCAGCAATTCTAATCCACTTTGGGTTTTCAGCTGTTTTTACATCTAAAGCAATAGACTAGAACTGAATTATCTTCTACATAGTAAAATCACAATTGTGGAATTACAGGAATTCTGGTGATATTAAGGTGAAATAACAAAACACAAAAGGCCCTATTTTAACAGTTGATGTGACAGTAAGTTTTAATAGAACCTGTAACTTCATTTTGGAAATGCTTCTCCACCAAATAAGGGCTTTTTCCCCTATTTAAGGAGCCAGATGGATTGAAAGATGTGGAAATAGGCAGCTGTAGATCTTGATCTTCCAGGTACCCCATGTACCTTTATTGAGCTTAATTATAATACTGTCAAATTGCCACGATCTCACTAAAGGATTTCTATTTGCTGTCAGTTAAAAATAAAGCCCTAAATACATTTTTATTCTTTCTACTGAGGGCATTGTCTGTTTTCTTTGTAAATGCCGTACAATAAACAAATTATTTAATAACCTAAAAAAAAAAAAAAAAAAA

1. Mutant SRPK1 gene 3’ UTR sequence (mutated miR-659-3P targeting sequences are marked blue)

GCCCCTGCCCAGCACCACAGCAGAGATCACACACTATGTTCATGCCCTTCCCCTTCAAGCATTTTCCTCTTCCCTTTTCAGGGTGAAGCTCTTCCTTCAAGAGTTTCTAGATCTTGTTTTTTTTTTAATCCAACATGTTCATTTGGGTTTGCTTACTTGACCCTGTGGAGATCCCCACAGCCATTGGGCATCCTAGGTGAATTTGGCCTTGGTTGGGCTCTGCCAAAGACTAATGGACTAAAATGTGAAACAGCCTCTTGCCCTGTACCTTTCCTTCCCATTAGGACATCCTTTAAATTATAAGCATCCTTTTTGAAAAGAGCTATGAAGGTGTATGAGCCCATCCTTTTATTCATTGACTCTAAGAGTCAAATTTTCTAGTGCATATCCTATTGCCAGCATAAGGATGAGGAGGGGGAAAGGGTCTTAATTCTATGTACAGCAGAGACATTAAACTTGCTGTGTCCGGGCTGCATCATCTTCCTGGACTGTTTCTGTTGTTCTCTGTGTTCACATTTTTTCCTGCAACTTTTAAGCTACTGTCTTTTTTAAATAGCTATATGAACACCAAATTTGGGTACCATTTTATCACTGTTCAAAGCACTGTCAAATTCCTTTCATCCTTTAATAGTTAAGATCTTTGAATCTTCAGTCTGATTTTTAATGTAAGCAAAAACAGAACCATTGAATAGTAATTTCTTGAGAACCTCAGGTGTTCTATAAACAGTCCTTTCCTGTATGTCTTCTATTACCCTAAGACCAGAGTTATTTTGGTTGGTTGTTTTGTTTTATTTTTTGTTTTTGTATCCATGGCTGGCACTTTACTCATTGCACTTGAGTTTATTGCCCCATAACTAAAGGATCAGGATGATGGTAGAACGGAGATCTGGGTTTCAGAGCTTTCCCATTTAAGAAAAATAGATCTTGAGATTCTGATTCTTTTCCAAACAGTCCCCTGCTTTCATGTACAGCTTTTTCTTTACCTTACCCAAAATTCTGGCCTTGAAGCAGTTTTCCTCTATGGCTTTGCCTTTCTGATTTTCTCAGAGGCTCGAGTCTTTAATATAACCCCAAATGAAAGGGTACCTGGGAGGGGTGGGATGGCACTTTTTTTTGTTGGTCTTGTTTTGTTTTGTTTTTTGGTTGGTTGGTTCGTTATTTTTTAAGATTAGCCATTCTCTGCTGCTATTTCCCTACATAATGTCAATTTTTAACCATAATTTTGACATGATTGAGATGTACTTGAGGCTTTTTTGTTTTAATTGAGAAAAGACTTTGCAATTTTTTTTTTAGGATGAGCCTCTCCTAGACTTGACCTAGAATATTACATATTCCTCCAGTAAGTAATACTGAAGAGCAAAAGAGAGGCAGGATTGGGGTCACAGCCGCTTCTTCAGCATGGACCAAGTGGGCCTTGGGGATTGCAGCGTTCTCGAAGTGGCTGTAGGACTCGAATTTACAGAAAGCCACAGAGGTGCAACTTGAGGCTCTGCTAGCAAGCCACCAGTGAGGCTATTGGGTAACCACCTTTCTATACAGGAGATTGGAATCTACTTTGTCATTTATCCACCACAGTGACAAAGGAAAAGTGGTGCCGTTATGCAATCCATTTAACTCATAAACATATTACTCTGAGTAACTGGCCAGCCATTCATCGGATCCTTCATTGGGTACTCCTGAAATCAGACATGTTCCTGTAGAAAGAATTTTAAGTTAGGCTTTCTATGCACCTATCAAGAATCAAGAGAATAGATTGTATCAAACAACGGCAGGGAAATCCTTCAGCAATTCTAATCCACTTTGGGTTTTCAGCTGTTTTTACATCTAAAGCAATAGACTAGAACTGAATTATCTTCTACATAGTAAAATCACAATTGTGGAATTACAGGAATTCTGGTGATATTAAGGTGAAATAACAAAACACAAAAGGCCCTATTTTAACAGTTGATGTGACAGTAAGTTTTAATAGAACCTGTAACTTCATTTTGGAAATGCTTCTCCACCAAATAAGGGCTTTTTCCCCTATTTAAGGAGCCAGATGGATTGAAAGATGTGGAAATAGGCAGCTGTAGATCTTGATCTTCCAGGTACCCCATGTACCTTTATTGAGCTTAATTATAATACTGTCAAATTGCCACGATCTCACTAAAGGATTTCTATTTGCTGTCAGTTAAAAATAAAGCCCTAAATACATTTTTATTCTTTCTACTGAGGGCATTGTCTGTTTTCTTTGTAAATGCCGTACAATAAACAAATTATTTAATAACCTAAAAAAAAAAAAAAAAAAA
